# PW01-006 – The effect of colchicine on physical growth in FMF

**DOI:** 10.1186/1546-0096-11-S1-A59

**Published:** 2013-11-08

**Authors:** N Cakar, TC Yoldas, B Acar, N Uncu, S Cayci

**Affiliations:** 1 nephrology and rheumatology, Turkey; 2 Ministry of Health Ankara Childrens Hospital, Ankara, Turkey

## Introduction

Familial Mediterranean fever is the most periodic fever sendrome and characterized by reccurent fever, abdominal pain, chest pain, arthritis/ arthralgia and erizipel like rash and self limited attacks with fever and serozal inflomation also elevated acute phase reactants. As with other chronic inflammatory diseases in patients with AAA growth may be negative effects.

## Objectives

The aim of the study was to evaluate the growth parameters after treatment with colchicine in patients with newly diagnosed by examining prepubertal, along with other identifying characteristics of the disease, taking into account the status of inflammation subclinic to investigate the effects on the growth of disease severity and activity.

## Methods

In the study, patients’ disease activity, attack frequency, colchicine dose, laboratory studies of control, height and weight which measured with a standard stadiometre, were recorded by a standard person. Growth rates detected by determining the height and weight SDS 's (Z-score) with an interval of six months. All measurements and evaluations were made on 51 prepubertal patient.

## Results

Patients, 22 (43.1%) were male and 29 (56.9%) were female with age of disease onset 5 (1-10) years, age of diagnosis and onset of therapy were 6.4 (1.3 to 11) years. According to Tel Hashomer criteria, severity score of the disease was 7 (4-10). When compered of height and weight of the patients at the beginning and at the one year of colchicine treatment a significant increase was found (p<0,05). Height SDS 's increased from -0.6 to -0.2; weight SDS' s increased from -0.6 to -0.4. The participants’ BMI SDS didn’t showed a significant increase, both the weight and height SDS proceedings of the increase was associated with, so it is possible there was no significant increase. The patients’, who had no episode, height and weight SDS increased more than who had episode. There was no effect of disease severity on growth (p> 0.05). Also height gain was not affected from elevated acute phase reactans at the attack free period, weight gain did. Body weight and body surface of the patients receiving doses of colchicine were averaged 0.03 ± 0.02 mg / kg / day was 0.98 ± 0.045 mg/m^2^. Colchicine dose was the same in patients with or without any attack.

## Conclusion

In this study, the positive effects of colchicine on the growth in prepubertal patients with newly diagnosed AAA was found. The prevention clinical episodes was enough for height growth but the prevention clinical episodes and subclinical inflammation was necessary for increase of weight.

## Disclosure of interest

None declared

